# Trends and clinicopathological characteristics of oral squamous cell carcinomas reported at a tertiary cancer hospital in Nepal during 1999 to 2009

**DOI:** 10.1002/cre2.278

**Published:** 2020-01-12

**Authors:** Rashmi Gajurel, Dej Kumar Gautam, Chin Bahadur Pun, Hari Prasad Dhakal, Beáta Éva Petrovski, Daniela Elena Costea, Dipak Sapkota

**Affiliations:** ^1^ Centre for International Health University of Bergen Bergen Norway; ^2^ Gade Laboratory for Pathology and Center for Cancer Biomarkers CCBio, Department of Clinical Medicine University of Bergen Bergen Norway; ^3^ Department of Oral Biology, Faculty of Dentistry University of Oslo Oslo Norway; ^4^ Department of Surgical Oncology, B.P. Koirala Memorial Cancer Hospital Bharatpur Nepal; ^5^ Department of Pathology, B. P. Koirala Memorial Cancer Hospital Bharatpur Nepal; ^6^ Department of Pathology and Laboratory Medicine, Nepal Cancer Hospital and Research Centre Lalitpur Nepal; ^7^ Department of Faculty Administration, IT‐section, Faculty of Dentistry University of Oslo Oslo Norway; ^8^ Department of Pathology Haukeland University Hospital Bergen Norway

**Keywords:** epidemiology, Nepal, oral cancer, oral squamous cell carcinoma

## Abstract

**Objective:**

Reliable data describing the trends and clinicopathological characteristics of oral squamous cell carcinoma (OSCC) in the Nepalese population are very limited. The current study aimed to examine the demographics, trend, and clinicopathological characteristics of OSCC reported to the main referral/tertiary cancer hospital, the B.P. Koirala Memorial Cancer Hospital (BPKMCH) in Nepal for a period of 11 years (1999–2009).

**Material and methods:**

This is a cross‐sectional study. Data were retrieved retrospectively from hospital register maintained in the Department of Ear, Nose, Throat at BPKMCH, categorized into demographic and clinicopathological variables and SPSS (V25) was used for the analysis.

**Results:**

In a period of 11 years, 3,452 cases of head and neck cancer were registered at the Department of Ear, Nose, Throat, BPKMCH. Out of 1,111 oral cancer cases, 1,081 (97.3%) were OSCC. A trend for increasing number of OSCCs presenting to BPKMCH was observed during that period. OSCC was found to be more common among males (73.0%), Brahmin/Chhetri ethnic groups (33.0%), in age group of 51–60 years (31.9%), and in Terai region (62.0%). Tongue (42.8%) was the most common site, followed by buccal mucosa (27.2%). Nevertheless, when stratified with respect to the geographical location and ethnicity, buccal mucosa was the most common site for OSCC in Terai region (63.9%, *p* = .002) and in Madhesi ethnic group (34.2%, *p* < .001). Majority of OSCC cases were diagnosed at advanced stage (49.7%, Stage IV) and received a combination therapy (42.0%).

**Conclusions:**

Hospital‐based records can provide valuable information on disease characteristics in countries like Nepal. This study revealed that the clinicopathological characteristics of OSCC in Nepal follow the global trend. Nevertheless, relationship between specific intraoral sites for OSCC with geographic location and ethnic groups is an interesting observation and requires further population‐based studies to clarify these findings.

## 1 INTRODUCTION

Oral cancer (OC), one major group of head and neck cancer, is a highly aggressive malignant tumor arising from the mucosal lining of the anterior two thirds of tongue, buccal mucosa, gingiva, floor of the mouth, and hard palate (Barnes, Eveson, Reichart, & Sidransky, 2005). Combined with lip and pharyngeal cancers, OC ranks as the seventh most common cancer type worldwide and accounts for approximately 300,000 deaths every year (Shield et al., 2017). Histologically, more than 90% of OCs are oral squamous cell carcinomas (OSCC; Sanderson, Wei, & Ironside, 2002). Despite improvement in the diagnostic and treatment modalities, the 5‐year survival of OSCC patients is around 60% in the high‐income countries (HICs), whereas the survival rate ranges from 23–57% in low‐ and middle‐income countries, including Nepal (Chen et al., 2018; Sankaranarayanan, Black, Swaminathan, & Parkin, 1998; Warnakulasuriya, 2009).

The incidence of OC is much higher in the low‐ and middle‐income countries as compared with the HICs (World Health Organization [WHO], 2018), and this is largely attributed to the differences in the prevalence and type of risk/habitual factors found in these geographic locations (Dhillon et al., 2018; Feller & Lemmer, 2012). For example, smokeless tobacco (gutka and betel quid) and alcohol are main risk factors in Southeast Asia; whereas smoking, alcohol, and recently the infection with human papillomavirus are important factors in western countries (Gillison, Chaturvedi, Anderson, & Fakhry, 2015; Joshi, Dutta, Chaturvedi, & Nair, 2014).

According to a recent report from GLOBOCAN (2018), OC combined with lip cancer is estimated to be the sixth leading cancer type in Nepal and the fourth most common cancer among males (International Agency for Research on Cancer [IARC], 2018). More alarming, the incidence is expected to increase significantly in the coming years (Cheong et al., 2017). However, there are very few hospital‐based studies conducted in Nepal, and majority of them suggest that head and neck cancer might be among the most common cancer types in the country (Bhatt et al., 2009; Pun, Pradhananga, Siwakoti, Subedi, & Moore, 2015). These observations indicate that the burden of OC in Nepal can in fact be much higher than estimated by the GLOBOCAN (2018).

Although a small country in area, Nepal is rich in diversity of ethnic groups. Majority of the ethnic groups differ from each other with respect to geographic location, sociocultural, and risk factors for OC and at the genetic level (Bennett, Dahal, & Govindasamy, 2008; Cole et al., 2017). For example, smokeless tobacco (gutka and betel quid) is more common in Madhesi ethnic group, residing mostly in the terai region. On the other hand, local (homemade) alcohol is consumed more commonly by Janjati (Mangoloid and Matwali) and Newar ethnic groups. Mangoloids are main inhabitant of mountains whereas Newars mostly reside in hilly regions (Bhatta, 2017; Dhital, Subedi, Gurung, & Hamal, 2001). This emphasizes the urgent need of studies aimed at mapping the epidemiological status of OC/OSCC in the country, taking into consideration the diverse population of the country. The current study aimed to examine the sociodemographics, trend, and clinicopathological characteristics of OSCC reported to the main referral/tertiary cancer hospital, the B.P. Koirala Memorial Cancer Hospital (BPKMCH) in Nepal for a period of 11 years (1999–2009).

## 2 STUDY CENTER AND DATA RETRIEVAL: METHODOLOGY

Ethical approval and permission for the current study were obtained from the Nepal Health Research Council (Approval Number 2283) and BPKMCH, respectively. This is a cross‐sectional study. Data were retrieved retrospectively from the register maintained in the Department of Ear, Nose, Throat, BPKMCH, Chitwan, Nepal, for a period from 1999 to 2009. BPKMCH is equipped with modern infrastructure and trained manpower and offers comprehensive management of cancer patients from Nepal as well as some from northern part of India. Over a period of 10 years, more than 48% cancer cases in Nepal were diagnosed at BPKMCH, the highest among all hospitals contributing to hospital‐based cancer registry in the country (BPKMCH, 2017).

**Figure 1:**
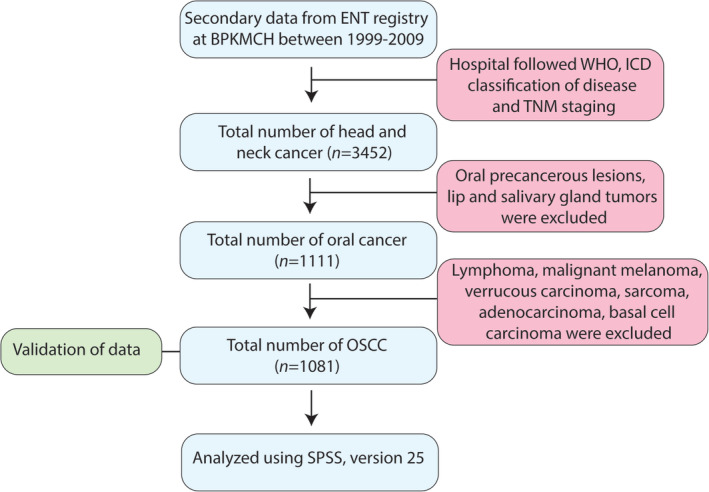
Flow chart showing steps in data collection and analysis

The retrieved data were validated by manually examining the hard copies of patient record files at BPKMCH. The hospital registry followed International Classification of Diseases‐10 classification of OC (WHO, 2004 2004) and the WHO‐TNM staging for stating the clinical stage of the tumor (IARC, 2019). Steps in data retrieval and analysis are summarized in (Figure 1). Briefly, precancerous oral lesions and other cancer types in the oral cavity (such as basal cell carcinoma, verrucous carcinoma, lymphoma, malignant melanoma, and adenocarcinoma) were excluded from the study. Out of 1,111 OC, 1,081 OSCC cases were used for analyses in the study.

### 2.1 Categorization of variables

Registered data were classified into the following demographic and clinicopathological variables:

1. Key demographic variables

- Gender: male, female; age: below 40, 41–50, 51–60, and above 60 years; nationality: Nepali, Indian.
- Ethnic groups: Brahmin/Chhetri, Madhesi, Janajati, and others (Dalit, Newar, Muslim, and Indian; Bennett et al., 2008).
- On the basis of the registered home address, patients were classified into three geographical areas: mountain (northern most part with an elevation that ranges from 4,877 to 8,848 m above the sea level), hills (located south to the mountain region with an elevation of 610 m above the sea level), and Terai (southernmost part that is flat and most densely populated; Central Bureau of Statistics [CBS], 2018). Patients from India were placed under category “others.”

Clinicopathological variables

- On the basis of the primary site of involvement, OSCC cases were divided in the following major anatomical sites: tongue, buccal mucosa, floor of the mouth, gingiva, palate, retromolar area, and alveolus. According to the surgical team involved in the biopsy/treatment, categorization of cases occurring in labial vestibule, buccal vestibule, and buccal mucosa was not consistent, and the majority of cases were grouped under “buccal mucosa.” As OSCC is very common in labial and buccal vestibules (where patients keep smokeless tobacco in the mouth), “buccal mucosa” in fact mostly refers to OSCC arising in labial and buccal vestibules.
- On the basis of (TNM) staging, OSCCs were categorized into Stages I, II, III, or IV.
- On the basis of the type of treatment given, patients were categorized into surgery, radiotherapy, chemotherapy, combination therapy (patient receiving more than one therapy), supportive/palliative, treatment not done, and missing data.

### 2.2 Management of missing variables and statistical analysis

Multiple imputation analysis was used to calculate the effect of missing data in the current study and to reduce the possible bias created by the missing variables. Multiple imputation replaces missing values with the imputed ones and gives reasonable assurance that the values being replaced with the imputation process are appropriate and match the other data not missing (by predicting what the actual value may have been). All seven variables (gender, age, ethnicity, geographical distribution, primary site of involvement, staging, and treatment modalities) were analyzed for missingness, out of which 22.2% incomplete data existed for two variables. Almost a fifth (19.6%) of the subjects/cases (212) had missing values in at least one data point, and 2.2% percent of all values had incomplete data.

Statistical analysis was done using Statistical Package for Social Sciences (Version 25 for Windows). Chi‐square test was used to examine the relationship between two variables.

## 3 RESULTS

**Figure 2:**
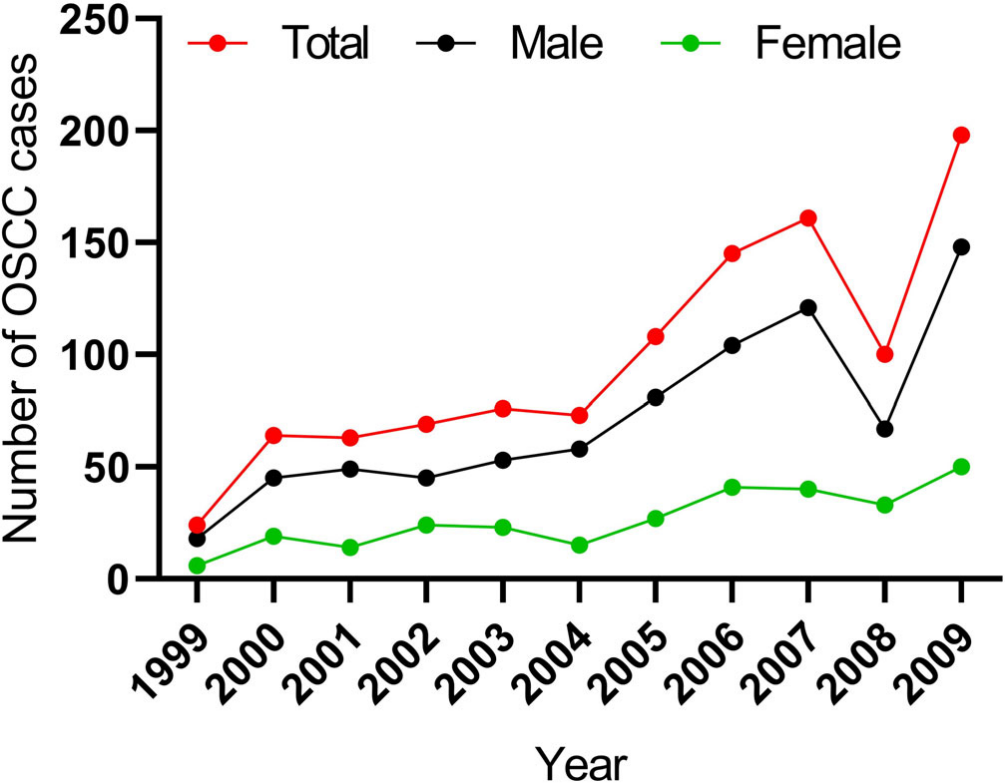
Increasing trends of oral squamous cell carcinoma (OSCC) cases at B.P. Koirala Memorial Cancer Hospital during 1999–2009. Increasing numbers of OSCC cases were registered at B.P. Koirala Memorial Cancer Hospital for both males and females with a slight decrease in 2008

In a period of 11 years, 3,452 cases of head and neck cancer were registered at the department of Ear, Nose, Throat, BPKMCH. Out of these, 1,111 were OCs. Approximately 97% (1,081) of OCs were OSCCs (Figure 1). During that period, a trend for increasing number of OSCCs presenting to BPKMCH was found for both males and females (Figure 2). It is, however, interesting to note a sharp decline in the number of OSCCs presented to BPKMCH in 2008. The occurrence of OSCC was found to be higher in males (73.0%) than in females (27.0%; male to female ratio: 2.7:1). Two thirds of patients belonged to the age group of 51–60 years (31.9%), followed by >60 years (28.7%), 41–50 years (21.6%), and < 40 years (17.8%). With respect to ethnicity, Brahmin/Chhetri (33.0%) were the most affected groups, followed by Madhesi (26.0%) and Janajati (22.0%). The geographical distribution of OSCC showed that 62.0% of patients were from Terai, 30.0% from hills, 1.5% from mountains, and 6.5% were from India.

On the basis of the primary site of involvement, tongue was the most common site (42.8%), followed by buccal mucosa (27.2%), floor of the mouth (13.8%), palate (6.5%) and gingiva (6.3%), retromolar area (2.6%), and alveolus (0.8%). Nevertheless, when stratified with respect to the geographical location and ethnicity, buccal mucosa was the most common site for OSCC in Terai region (63.9%, *p* = .002) and in Madhesi ethnic group (34.2%, *p* < .001; Tables 1 and 2). Almost half (49.7%) of the OSCCs were found to be diagnosed at late stage (Stage IV), followed by Stage III (18.3%). Only a small number of patients were diagnosed at Stage II (10.6%) and Stage I (2.7%). For 18.7% of the cases, data were missing for disease stage (Table S1).

**Table 1:** Distribution of location of oral squamous cell carcinoma cases with respect to ethnicity of oral squamous cell carcinoma patients

OSCC sites	Brahmin/Chhetri n (%)	Janajati n (%)	Madhesi n (%)	Others n (%)	Total n (%)	*p* value
Tongue	175 (38.4)	123 (27.0)	85 (18.6)	73 (16.0)	456 (100.0)	<.001
Buccal mucosa	93 (31.9)	36 (12.3)	100 (34.2)	63 (21.6)	292 (100.0)	
Floor of the mouth	36 (24.2)	49 (32.9)	39 (26.2)	25 (16.7)	149 (100.0)	
Others^a^	55 (31.6)	30 (17.2)	52 (29.9)	37 (21.3)	174 (100.0)	
Total					1,071 (100.0)	

Abbreviation: OSCC, oral squamous cell carcinoma.

a Others include OSCC in alveolus, retromolar area, palate, and gingiva.

**Table 2:** Distribution of location of oral squamous cell carcinoma cases with respect to geographical location of OSCC patients registered at B.P. Koirala Memorial Cancer Hospital

OSCC sites	Mountain n (%)	Hill n (%)	Terai n (%)	Others n (%)	Total	*p* value
Tongue	4 (0.8)	166 (35.9)	270 (58.3)	23 (5.0)	463 (100.0)	<.002
Buccal mucosa	7 (2.4)	67 (22.8)	188 (63.9)	32 (10.9)	294 (100.0)	
Floor of the mouth	2 (1.3)	46 (30.9)	93 (62.4)	8 (5.4)	149 (100.0)	
Others^a^	3 (1.7)	45 (25.7)	119 (68.0)	8 (4.6)	175 (100.0)	
Total					1,081 (100.0)	

Abbreviation: OSCC, oral squamous cell carcinoma.

a Others include OSCC gingiva, palate, retromolar area, and alveolus.

Almost half (42.0%) of the patients received combination therapy (i.e., surgery, radiotherapy, or chemotherapy) for the treatment of OSCC lesions. Only 16.7% OSCCs were treated with radiation only, 14.1% with chemotherapy, and 4.4% with surgery. Few patients (1.9%) received palliative/supportive treatment, 14.2% did not receive any treatment, whereas for 6.7%, data were missing on treatment received. Late stage OSCCs were significantly related with combination therapy (*p* < .0001; Table S2). Additionally, significant positive relation was found between stage and age (*p* = .031) and stage and male sex (*p* = .003; Table S2).

Information on risk habits for OSCC were missing in more than 20% of the cases and were therefore excluded from the study.

## 4 DISCUSSION

Epidemiological study of a disease provides key information that can be used for understanding the etiology of the disease and to plan preventive and management strategies (Gracia et al., 2017). This study provides descriptive epidemiology of OSCC and its characteristics in a referral cancer hospital in Nepal from 1999 to 2009.

The present study revealed a trend for increasing number of OSCCs presenting to BPKMCH over the study period in both males and females (Figure 1). Several factors might have contributed to this observation. First and importantly, during the earlier years of data collection, the hospital was in its earlier days of service (Nepal Law Commission, 1997) and therefore the patients' attendance to the hospital might have been lower. Second, higher level of patient awareness for the disease and increased accessibility to BPKMCH during the later years might have resulted in higher number of OSCC patients attending BPKMCH. Despite of the introduction of Framework Convention on Tobacco Control (tobacco law) in Nepal, the tobacco control has been ineffective due to limited resources and poor implementation, especially in the rural areas of Nepal (WHO, 2008, 2011). Indeed, increased availability and accessibility to risk factors such as smokeless tobacco (Subedi & Sharma, 2013) might also have contributed to the increasing numbers of OSCCs reported to BPKMCH.

A moderate decline in OSCCs was seen in 2008. This might be related to a low patient attendance to hospital due to insurgency and political crisis in the country during that year. Most of the transport systems had been affected due to frequent strikes and curfews (British Broadcasting Corporation, 2008). This is consistent with a report from the same hospital that showed an increasing trend for cancer cases in Nepal from 2003 to 2012 with slight drop in 2008 (Poudel, Huang, & Neupane, 2016).

OSCC was more frequent in males than in females, similar to a recent report by GLOBOCAN (IARC, 2018) and studies from the neighboring country India (Malhotra et al., 2014; Salian, Dinakar, Shetty, & Ajila, 2016). The higher number of OSCCs among males might be related to higher percentage of males using tobacco products (betel quid and tobacco chewing) and alcohol than women, as suggested by previous reports (Lee et al., 2011).

An explanation for the link between males and late stage OSCC as observed in the current study is currently unknown. However, one cannot rule out the possibility that men give less priority to oral health than women, thereby seeking medical attention late in the disease process (Nimako‐Boateng, Owusu‐Antwi, & Nortey, 2016).

The risk of developing OSCC increases with age, and this disease is considered as a neoplasm of fifth decade(Barnes et al., 2005). In line with this, the mean age for OSCCs was 55 years in our study, an age group considered as a risk group of OC (Zini, Czerninski, & Sgan‐Cohen, 2010). This result is in accordance with several other studies from different demographic groups (Alves et al., 2017; Malhotra et al., 2014).

In the present study, the most common site for OSCC was tongue followed by buccal mucosa. Although similar to global reports (Feller & Lemmer, 2012; Pires et al., 2013), this observation is somewhat surprising given the fact that smokeless tobacco habit, where the tobacco product is kept in contact with the epithelium in the buccal/labial vestibule, is a common practice in Nepal (Subedi & Sharma, 2013). However, when stratified with respect to geography, 63.9% of OSCC from Terai region were located in buccal vestibule. Indeed, smokeless tobacco habit is more prevalent in people from Terai as compared with the people in hills and mountains (Bhatta, 2017).

The present study revealed a higher occurrence of OSCC among Brahman/Chhetri as compared with other ethnic groups. Several factors might have contributed to this observation. First, Brahmans/Chhetris constitute the largest proportion of Nepalese population (CBS, 2018). Second, tobacco use has been suggested to be more common in Brahmans/Chhetris as compared with other ethnic groups (Raspanti et al., 2015). Third, difference in the genotypes among different ethnic groups might have contributed to a variable susceptibility to carcinogenesis (Özdemir & Dotto, 2017). Indeed, different ethnic groups in Nepal seem to have diverse genetic makeups (Cole et al., 2017).

The geographical distribution demonstrated that the highest number of OSCC patients were from Terai, followed by the hilly and mountain regions. A similar pattern has been suggested in previous reports (Bhatta, 2017). The high number of OSCCs in Terai region is likely because it is the most populated area among the three geographical regions (CBS, 2018). However, likely contribution of higher number OSCC cases associated with prevalent habit of smokeless tobacco and easy access to BPKMCH cannot be ruled out. This warrants more in‐depth population‐based studies to investigate/clarify the association between OSCC cases and geographical location.

In developing countries like Nepal, the diagnosis of OSCC at an advanced stage is common (Chettri, Bhandary, Singh, Sinha, & Karki, 2013; Malhotra et al., 2014). Likewise, more than two thirds of the patients were found to be at Stages III and IV in this study. The late presentation of OSCC is likely due to lack of awareness about the disease, high cost associated with treatment, and/or low priority given to oral health (Osman et al., 2010; Rogers, Vedpathak, & Lowe, 2011). Moreover, pain is not a common symptom of earlier stage OSCC, thereby delaying patients' attention to medical care (Cuffari, Siqueira, Nemr, & Rapaport, 2006). Treatment modalities of OSCC differ according to the primary site and cervical lymph node metastasis (Warnakulasuriya, 2009). Generally single treatment (usually surgery) is the preferred method for early‐stage cancers, whereas patients with advanced stage are treated with a combination of surgery and radiotherapy or chemotherapy (Prelec & Laronde, 2014). In parallel, a significant relation between the higher OSCC grade and combination therapy was observed in the current study.

Due to the improper/inadequate record keeping and maintenance of data, information on risk habits for OSCC were missing in more than 20% of the cases and were therefore excluded from the study. We understand that this is a major limitation of the study, given that different types of risk habits are specific‐to‐specific ethnic groups and geographical locations in Nepal. Moreover, the generalizability of the current findings is limited due to the fact the study is based on patient data from a single main referral cancer hospital, although BPKMCH is the main referral hospital for cancer in Nepal.

In conclusion, hospital‐based records can provide valuable information on disease characteristics and generate relevant hypothesis in countries like Nepal where properly conducted population‐based studies are limited. However, the record system needs to be optimized to minimize missing variables and to improve correctness of the data. This study revealed that the epidemiology of OSCC in Nepal follows the global trend. Of interest, tongue was identified to be the most common intraoral site, following thus the pattern of OSCC from the Western world rather than the pattern of OSCC in Southeast Asia. Additionally, relationships between specific intraoral sites for OSCC with geographic distribution and ethnic groups are interesting observation that require further population‐based studies to substantiate them.

## CONFLICT OF INTEREST

The authors declare that there is no conflict of interest.

## AUTHOR CONTRIBUTIONS

D. S., D. K. G., and D. E. C. conceived and designed research; D. K. G., C. B. P., H. P. D., and D. S. contributed with data; R. G., B. E. P., and D. S. performed the statistical analysis. R. G., D. E. C., and D. S. wrote the manuscript. All authors critically reviewed and approved the manuscript.

## Supporting information


**Table S1**. Frequencies of missing variables.
**Table S2.** Relationship between stage, age group, gender and treatment modalities for OSCC registered at B.P. Koirala Memorial Cancer Hospital, Nepal.
