# Supplementary material for: Trends and clinicopathological characteristics of oral squamous cell carcinomas reported at a tertiary cancer hospital in Nepal during 1999 to 2009
Source: Clin Exp Dent Res. 2020 Jan 12;6(3):356–62. doi: 10.1002/cre2.278 (PMC7301398; doi:10.1002/cre2.278)
Supplement: Supplementary file 1 — Table S1. Frequencies of missing variables. Table S2. Relationship between stage, age group, gender and treatment modalities for OSCC registered at B.P. Koirala Memorial Cancer Hospital, Nepal. [file CRE2-6-356-s001.docx]

**Supplementary Tables:**

Table S1. Frequencies of missing variables.

___________________________________________________

Missing variables Frequency (percentage)

________________________________________________

Ethnicity 10 (0.9%)

Stage 202 (18.7%)

Treatment Modalities 72 (6.7%)

Risk habits* 259 (24%)

___________________________________________________

*Risk habits were excluded from the study.

|  |  |
| --- | --- |

Table S2. Relationship between stage, age group, gender and treatment modalities for OSCC registered at B.P. Koirala Memorial Cancer Hospital, Nepal.

| Variables |  | Early stage n (%) | Late stage  n (%) | Total (%) | *p-value* |
| --- | --- | --- | --- | --- | --- |
| Age (years) |  |  |  |  |  |
|  | <40 | 39 (24.7) | 119 (75.3) | 158 (100.0) | ***0.031*** |
|  | 41-50 | 33 (17.3) | 158 (82.7) | 191 (100.0) |  |
|  | 51-60 | 43 (15.3) | 238 (84.7) | 281 (100.0) |  |
|  | >60 | 29 (11.6) | 220 (88.4) | 249 (100.0) |  |
| Gender |  |  |  |  |  |
|  | Male | 97 (15.0) | 550 (85.0) | 647 (100.0) | ***0.003*** |
|  | Female | 47 (20.3) | 185 (79.7) | 232 (100.0) |  |
| Treatment modalities |  |  |  |  |  |
|  | Monotherapy | 57 (20.9) | 216 (79.1) | 273 (100.0) | ***0.000*** |
|  | Combined therapy | 68 (16.5) | 344 (83.5) | 412 (100.0) |  |
|  | Others | 19 (9.8) | 175 (90.2) | 194 (100.0) |  |

Note: Early stage: stages I and II; Late stage: stages III and IV; Monotherapy: surgery, radio- or chemotherapy; Combination therapy: surgery + radio- and/or chemotherapy
